# Supplementary material for: External globus pallidus input to the dorsal striatum regulates habitual seeking behavior in male mice
Source: Nat Commun. 2023 Jul 12;14:4085. doi: 10.1038/s41467-023-39545-8 (PMC10338526; doi:10.1038/s41467-023-39545-8)
Supplement: Supplementary file 1 — Supplementary Information [file 41467_2023_39545_MOESM1_ESM.pdf]

External globus pallidus input to the dorsal striatum regulates habitual seeking behavior in male mice

Matthew Baker[1,#], Seungwoo Kang[1,2,#], Sa-Ik Hong[1], Minryung Song[3], Minsu Abel Yang[3], Lee Peyton[1], Hesham Essa[1], Sang Wan Lee[3,4], and Doo-Sup Choi[1,5,6,*]

[1]Department of Molecular Pharmacology and Experimental Therapeutics, Mayo Clinic, Rochester, Minnesota 55905, USA. [2]Department of Pharmacology and Toxicology, Medical College of Georgia, Augusta University, Augusta, Georgia 30912, USA. [3]Department of Brain and Cognitive Sciences, [4]Department of Bio and Brain Engineering, Korea Advanced Institute of Science and Technology (KAIST), Daejeon, 34141, Republic of Korea. [5]Department of Psychiatry and Psychology, [6]Neuroscience Program, Mayo Clinic College of Medicine and Science, Rochester, Minnesota 55905, USA.

[#]These authors contributed equally to this work.

[*]Corresponding author: choids@mayo.edu

Keywords: external globus pallidus, arkypallidal neuron, goal-directed behavior, habit, reward-seeking behavior

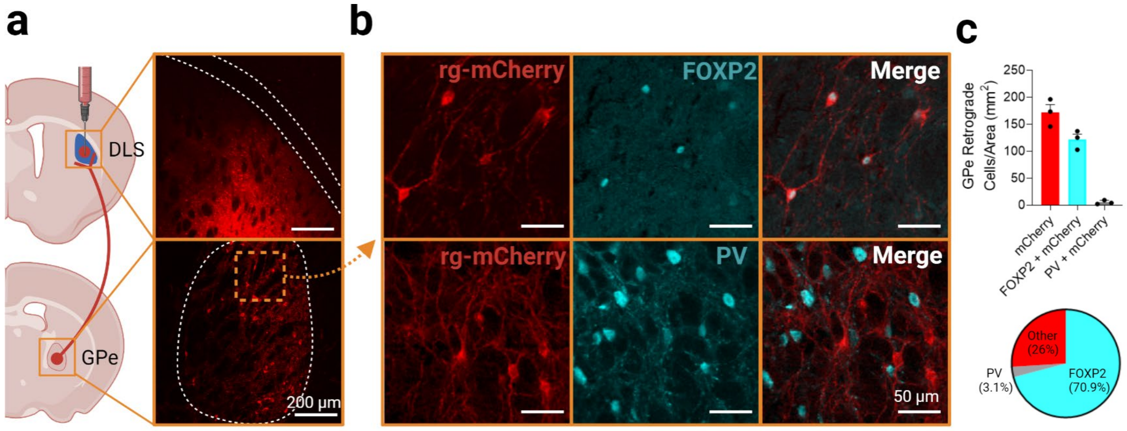

Supplementary Figure 1. Cellular markers of dorsolateral striatum (DLS)-projecting external globus pallidus (GPe) arkypallidal neurons. (a) Schematic of retrograde mCherry virus injection into the DLS and representative IHC images at the injection site and in the GPe. Scale: 200 µm. **(b)** representative IHC images of GPe arkypallidal cells and FOXP2 and PV cellular markers. Scale: 50 µm. **(c)** Quantification of overlap of retrograde mCherry with FOXP2 and PV cellular markers in the GPe. *n* = 3 mice/group. Data represent mean ± SEM. Source data are provided as a Source Data file. Supplementary Figure 1a was created with BioRender.com.

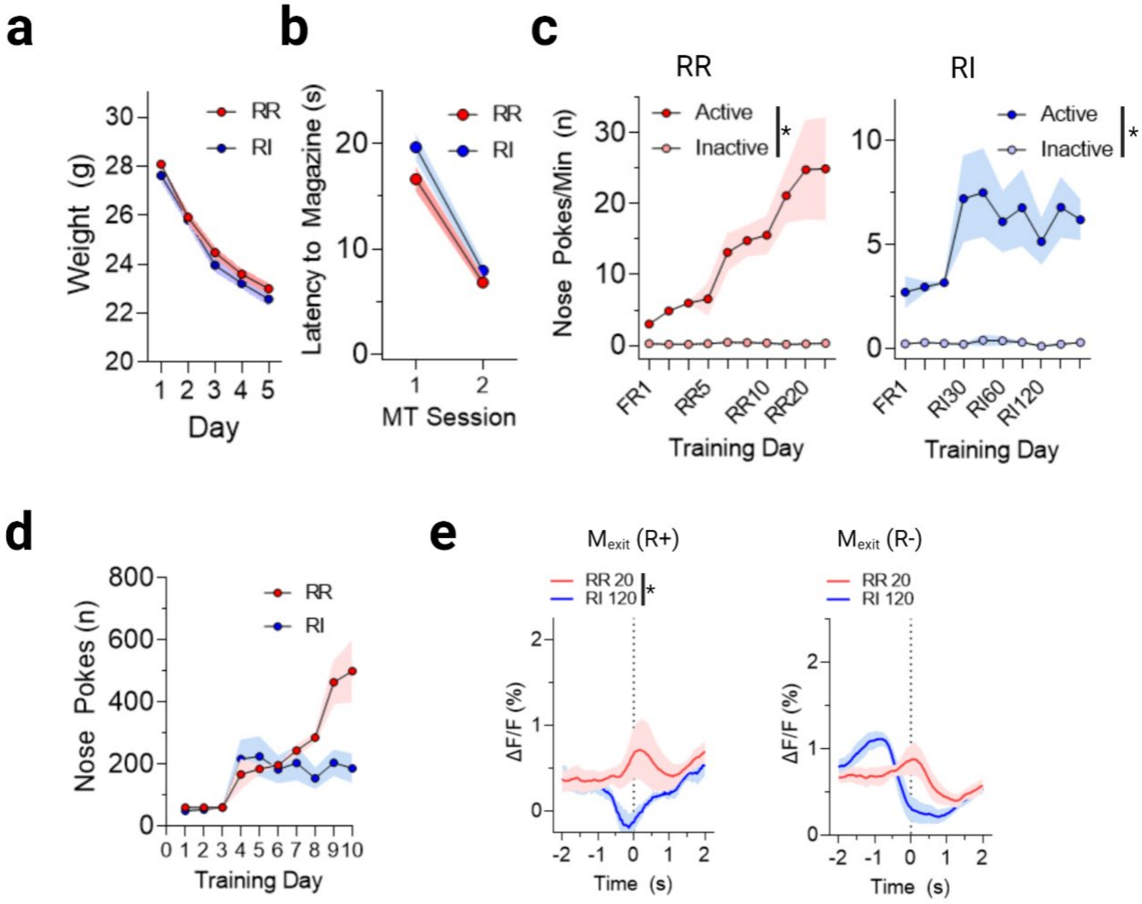

Supplementary Figure 2. External globus pallidus (GPe) arkypallidal Ca^{2+} imaging training. (a) Average weight of mice undergoing food restriction prior to operant conditioning. **(b)** Latency to magazine decreased across training sessions. $F_{(1,16)} = 142.9$, $p < 0.0001$ for time, $F_{(1,16)} = 5.34$, $p < 0.04$ for group, $F_{(1,16)} = 1.14$, $p = 0.30$ for interaction. *n* = 5 mice/group. **(c)** Nose poke rates increased over time in the active hole. For RR, $F_{(1,80)} = 127.3$, $p < 0.0001$ for nose poke, $F_{(9,80)} = 4.99$, $p < 0.0001$ for time, $F_{(9,80)} = 4.93$, $p < 0.0001$ for interaction. For RI, $F_{(1,80)} = 142.1$, $p < 0.0001$ for nose poke, $F_{(9,80)} = 1.85$, $p = 0.07$ for time, $F_{(9,80)} = 1.77$, $p = 0.09$ for interaction. **(d)** Total nosepokes for RR and RI groups across the training session. **(e)** Calcium signal for rewarded (R+) and unrewarded (R-) magazine exit. For the rewarded magazine exit [M_{exit} (R+)], $F_{(1,7)} = 6.66$, $p = 0.036$ for group (RR vs RI), $F_{(120,840)} = 0.85$, $p = 0.87$ for time, $F_{(120,840)} = 1.26$, $p = 0.039$ for interaction. For the non-rewarded magazine exit [M_{exit} (R-)], $F_{(1,7)} = 0.20$, $p = 0.67$ for group (RR vs RI), $F_{(120,840)} = 8.65$, $p < 0.0001$ for time, $F_{(120,840)} = 5.51$, $p < 0.0001$ for interaction. Two-way repeated measures ANOVA with Tukey's *posthoc* tests were used for (**b-e**). *n* = 5 (RR), 4 (RI). See supplementary Table 3 for specific significant time ranges. Data represent mean ± SEM. *$p < 0.05$. See Supplementary Table 2 for full statistical information. Source data are provided as a Source Data file.

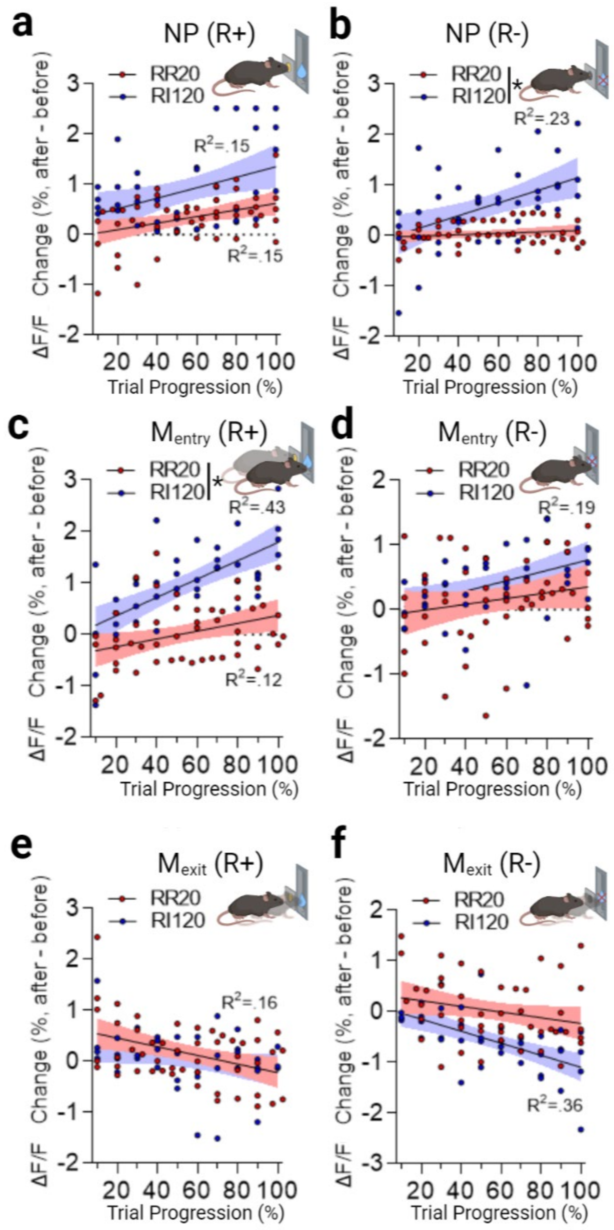

Supplementary Figure 3. External globus pallidus (GPe) arkypallidal neuron Ca^{2+} signal changes across an operant session. For rewarded nose poke **(a)** unrewarded nose poke **(b),** simple linear regression models for the association between a change in GPe arkypallidal neuron Ca^{2+} signal (2 sec after – 2 sec before) and operant trial duration averaged into 10 blocks. For the rewarded nose poke (NP+), $F_{(1,48)}$ = 8.26, p = 0.006 for RR, $F_{(1,38)}$ = 6.78, p = 0.013 for RI, $F_{(1,86)}$ = 0.79, p = 0.38 for RR versus RI. For the non-rewarded nose poke (NP-), $F_{(1,48)}$ = 1.48, p = 0.23 for RR, $F_{(1,38)}$ = 11.66, p = 0.002 for RI, $F_{(1,86)}$ = 10.08, p = 0.002 for RR versus RI. Rewarded magazine entry **(c)** unrewarded magazine entry (**d**). For the rewarded magazine entry [M_{entry} (R+)], $F_{(1,48)}$ = 6.62, p = 0.013 for RR, $F_{(1,38)}$ = 28.86, p < 0.0001 for RI, $F_{(1,86)}$ = 5.34, p = 0.023 for RR versus RI. For the non-rewarded magazine entry [M_{entry} (R-)], $F_{(1,48)}$ = 1.96, p = 0.17 for RR, $F_{(1,38)}$ = 9.08, p = 0.005 for RI, $F_{(1,86)}$ = 0.69, p = 0.41 for RR versus RI. Rewarded magazine exit **(e)** and unrewarded magazine exit **(f)**. For the rewarded magazine exit [M_{exit} (R+)], $F_{(1,48)}$ = 9.08, p = 0.004 for RR, $F_{(1,38)}$ = 3.12, p = 0.09 for RI, $F_{(1,86)}$ = 0.60, p = 0.44 for RR versus RI. For the non-rewarded magazine exit [M_{exit} (R-)], $F_{(1,48)}$ = 3.30, p = 0.075 for RR, $F_{(1,38)}$ = 21.77, p < 0.0001 for RI, $F_{(1,86)}$ = 2.99, p = 0.13 for RR versus RI. Linear regression tests were used for (**a-f**). Lines indicate regression line ± 95% CI. R^2 value indicates a significant association between the change in Ca^{2+} signal and behavioral event progression across a trial. *p < 0.05 for between-group slope comparisons. See Supplementary Table 2 for full statistical information. Source data are provided as a Source Data file. Supplementary Figure 3a-f were created with BioRender.com.

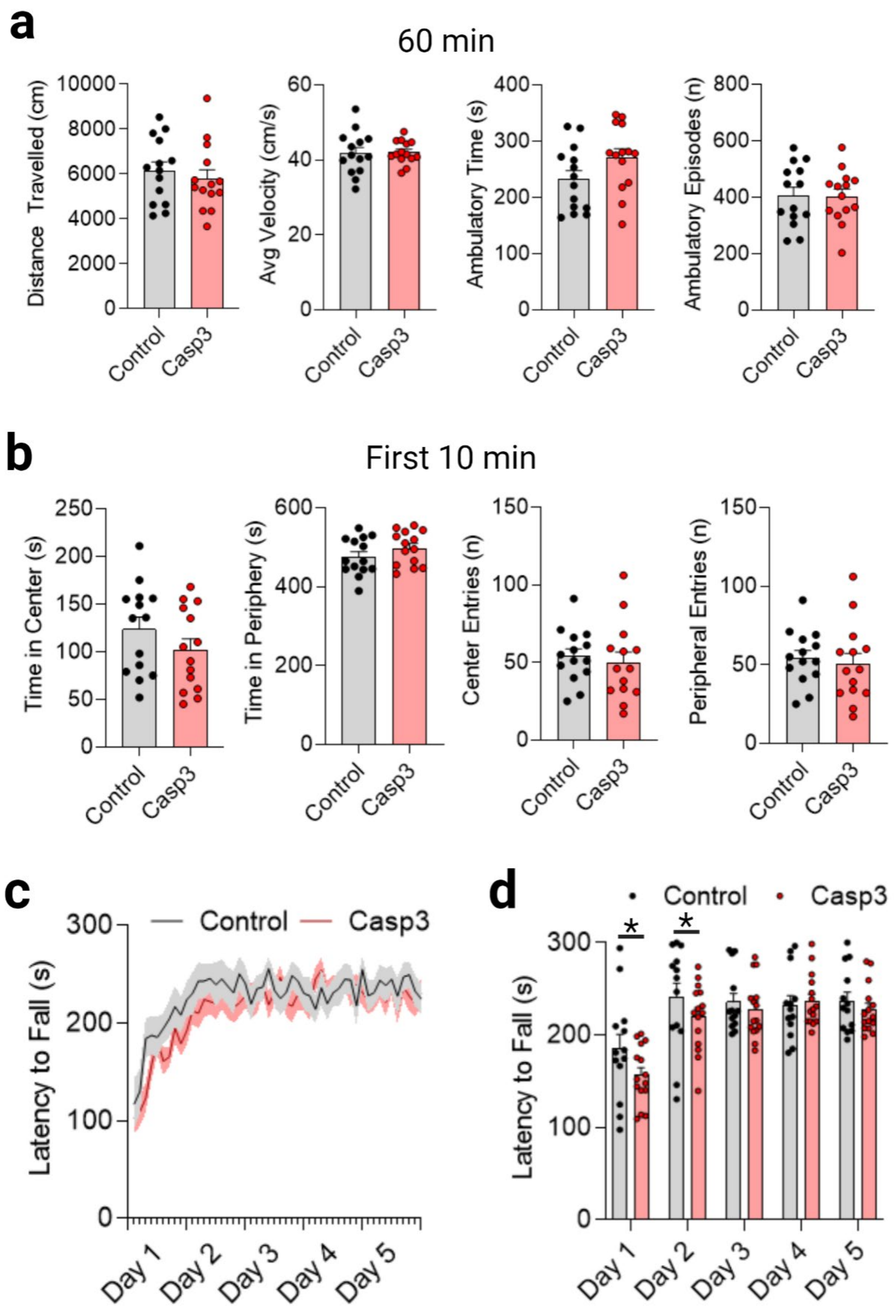

Supplementary Figure 4. Effects of caspase-3 (casp3) ablation of external globus pallidus (GPe) arkypallidal neurons on spontaneous locomotion and motor skill acquisition. (a) GPe arkypallidal neuron ablation did not alter distance traveled or average velocity in the open field test, **(b)** or anxiety-like behaviors during the first 10 minutes. **(c)** Latency to fall increased across training sessions for both groups. $F_{(49,1274)} = 8.68$, $p < 0.0001$ for time, $F_{(1,26)} = 1.28$, $p = 0.27$ for group (control vs casp3), $F_{(49,1274)} = 0.76$, $p = 0.89$ for interaction. **(d)** Caspase mice had a shorter latency to fall compared to control mice for days. $F_{(4,130)} = 58.82$, $p < 0.0001$ for time, $F_{(1,130)} = 1.49$, $p = 0.22$ for group (control vs casp3), $F_{(4, 130)} = 2.99$, $p = 0.018$ for interaction. Sidak's *posthoc* tests show *$p < 0.05$ in day 1 ($p = 0.0005$) and day 2 ($p = 0.018$). Two-tailed Mann-Whitney tests were used for (**a-b**). Two-way repeated measures ANOVA with Sidak's *posthoc* tests were used for (**c-d**). $n = 14$ mice/group. Data represents mean ± SEM. *$p< 0.05$. See Supplementary Table 2 for full statistical information. Source data are provided as a Source Data file.

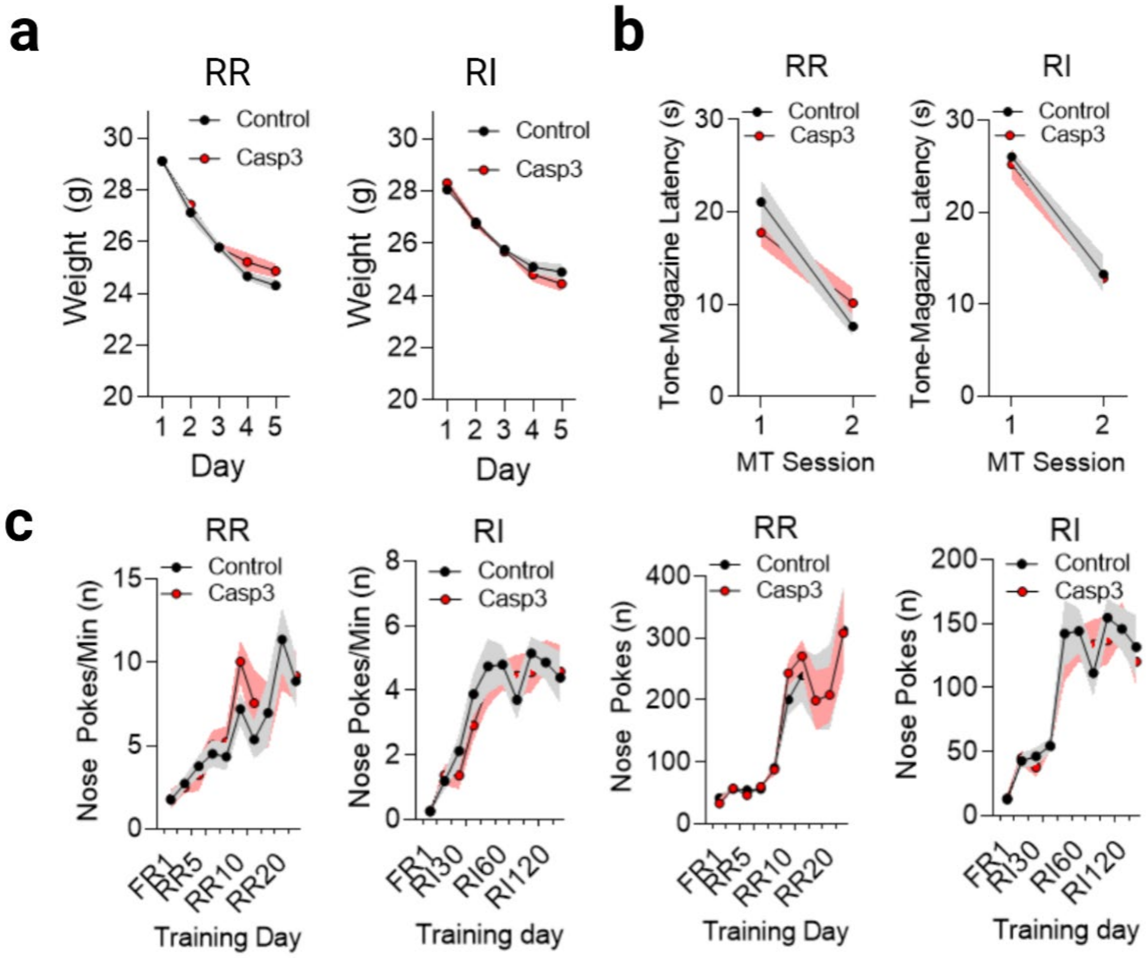

Supplementary Figure 5. Effects of caspase-3 (casp3) ablation of external globus pallidus (GPe) arkypallidal neurons on operant training. (a) Average weight of mice undergoing food restriction prior to operant conditioning. **(b)** Latency to magazine decreased across training sessions. For RR, $F_{(1,18)}$ = 29.91, p < 0.0001 for time, $F_{(1,18)}$ = 0.00, p = 0.99 for group, $F_{(1,18)}$ = 2.36, p = 0.11 for interaction. For RI, $F_{(1,18)}$ = 30.55, p < 0.0001 for time, $F_{(1,18)}$ = 0.13, p = 0.73 for group, $F_{(1,18)}$ = 0.01, p = 0.98 for interaction. **(c)** Nose poke rates increased over time in the active hole. For RR, $F_{(9,161)}$ = 13.38, p < 0.0001 for time, $F_{(1,18)}$ = 0.21, p = 0.65 for group, $F_{(9,161)}$ = 0.66, p = 0.74 for interaction. For RI, $F_{(9,161)}$ = 20.64, p < 0.0001 for time, $F_{(1,18)}$ = 0.28, p = 0.61 for group, $F_{(9,161)}$ = 0.52, p = 0.86 for interaction. Total nosepokes also increased over time. For RR, $F_{(9,161)}$ = 18.29, p < 0.0001 for time, $F_{(1,18)}$ = 0.03, p = 0.86 for group, $F_{(9,161)}$ = 0.2, p = 0.99 for interaction. For RI, $F_{(9,161)}$ = 25.26, p < 0.0001 for time, $F_{(1,18)}$ = 0.17, p = 0.68 for group, $F_{(9,161)}$ = 0.35, p = 0.96 for interaction. Two-way repeated measures ANOVA tests were used for (**b-c**). n = 10 mice/group. Total nosepokes across training days. Data represent mean ± SEM. See Supplementary Table 2 for full statistical information. Source data are provided as a Source Data file.

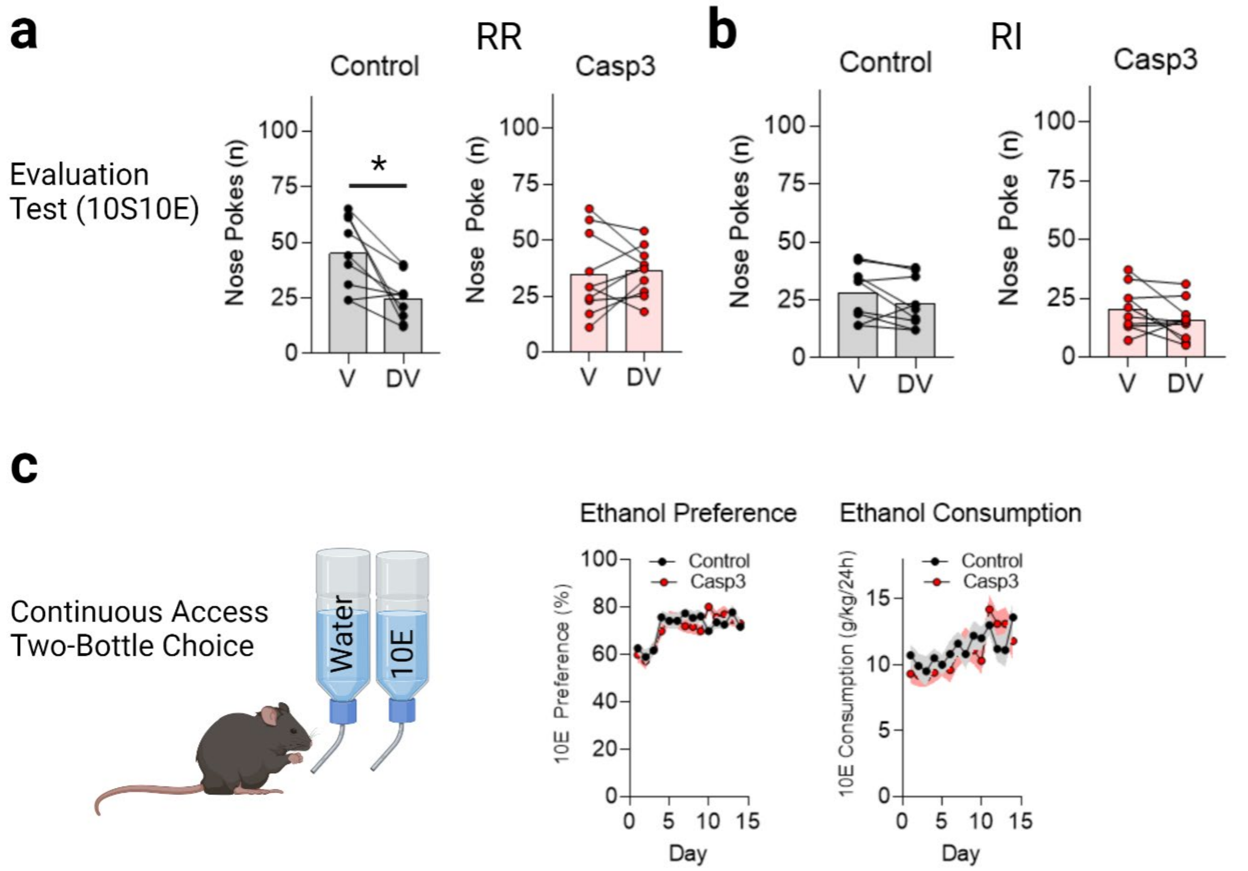

Supplementary Figure 6. Effect of external globus pallidus (GPe) arkypallidal neuron ablation (casp3) on goal-directed and habitual seeking of an ethanol-containing reward and baseline ethanol preference and consumption. (a) RR-trained (Goal-directed; 10% sucrose 10% ethanol reward) sham mice reduced nosepoke responses in the devalued state, confirming goal directed behavior. However, casp3 mice showed no changes in nose poke responses between valued and devalued states, typical of habitual behavior. **(b)** RI-trained mice showed no differences between valued and devalued states, suggesting habitual behavior. **(c)** 10% ethanol preference and consumption during a continuous access two-bottle choice test. For preference, $F_{(13,234)} = 9.09$, $p < 0.0001$ for time, $F_{(1,18)} = 0.33$, $p = 0.57$ for group, $F_{(13,234)} = 1.29$, $p = 0.22$ for interaction. For consumption, $F_{(13,234)} = 3.54$, $p < 0.0001$ for time, $F_{(1,18)} = 0.39$, $p = 0.54$ for group, $F_{(13,234)} = 0.87$, $p = 0.58$ for interaction. Wilcoxon test was used for (**a-b**). Two-way repeated measures ANOVA tests were used for (**c**). $n = 8$ mice/group. Data represent mean ± SEM. $^{*}p < 0.05$. See Supplementary Table 2 for full statistical information. Source data are provided as a Source Data file. Supplementary Figure 6c was created with BioRender.com.

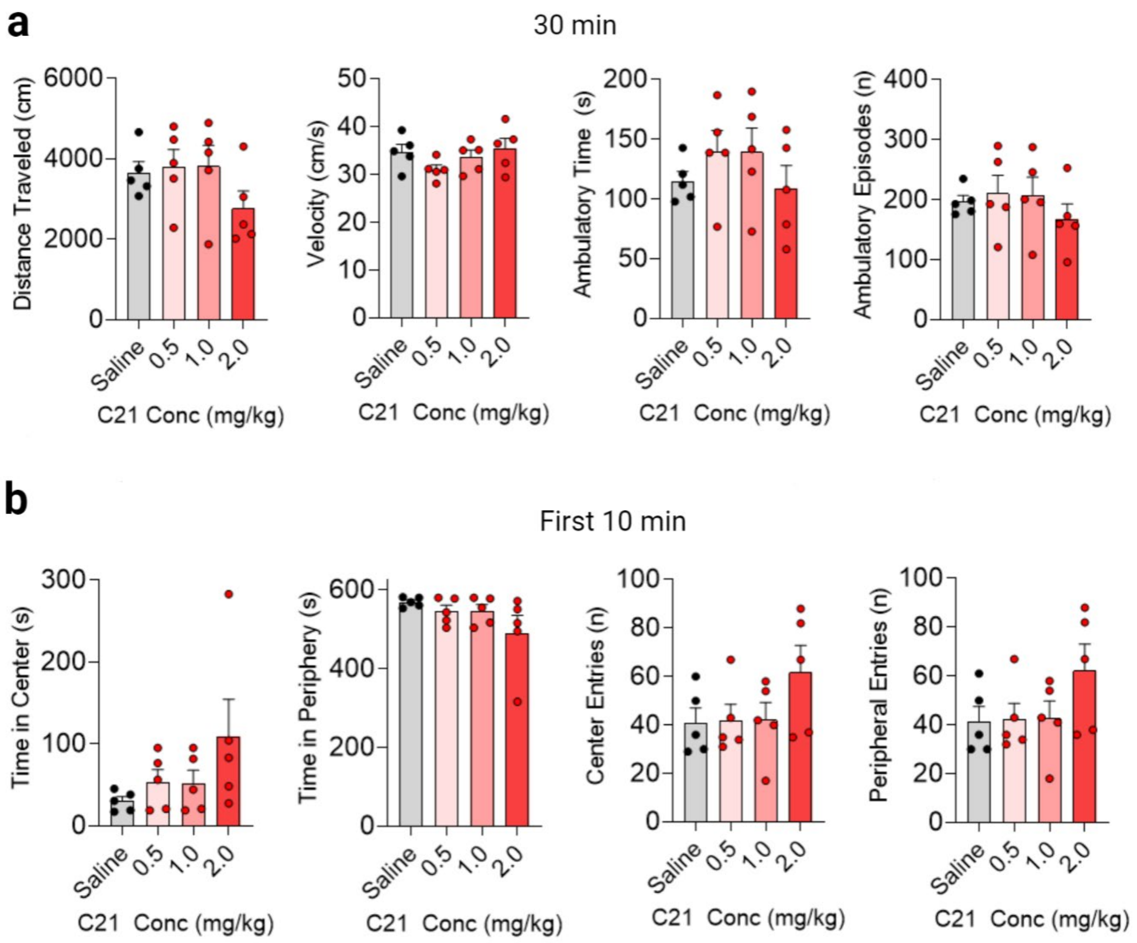

Supplementary Figure 7. Effects of C21 concentration on spontaneous locomotion in mice expressing hM3Dq in external globus pallidus (GPe) arkypallidal neurons in the open field test. (a) There was no effect of C21 concentration on distance traveled, average velocity, or ambulatory time and episodes. **(b)** Nor was there any effect on anxiety-like behaviors during the first 10 minutes of the open field test. Kruskal-Wallis tests were used for (**a-b**). *n* = 5 mice/group. Data represent mean ± SEM. See Supplementary Table 2 for full statistical information. Source data are provided as a Source Data file.

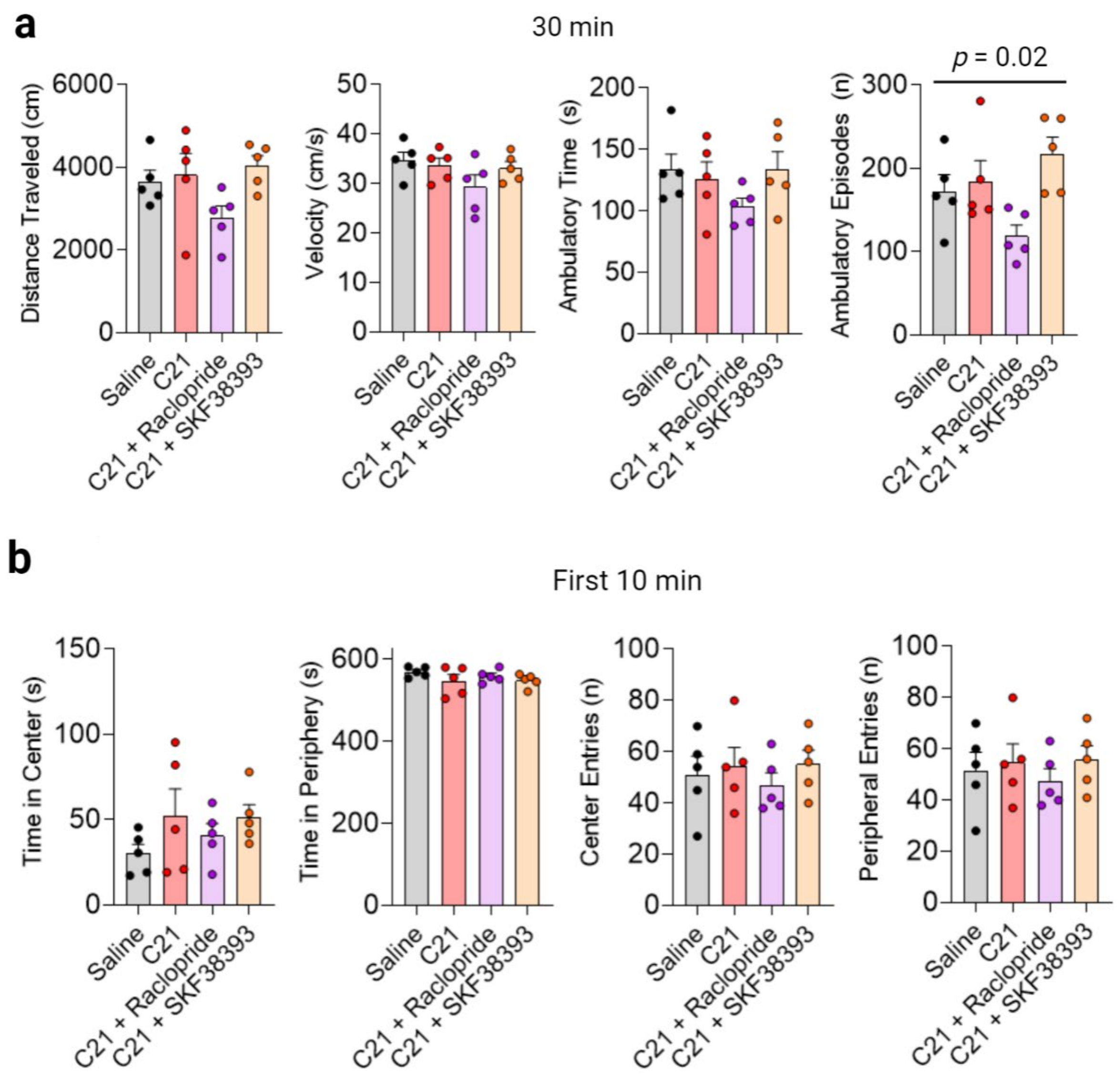

Supplementary Figure 8. Effects of IP injection groups on spontaneous locomotion in mice expressing hM3Dq in external globus pallidus (GPe) arkypallidal neurons in the open field test. (a) No effects of the injection group on distance traveled, average velocity, or ambulatory time, except the main effect on ambulatory episodes ($p = 0.02$). **(b)** No effects on anxiety-like behaviors during the first 10 minutes of the open field test. Kruskal-Wallis tests were used for (**a-b**). $n = 5$ mice/group. Data represent mean ± SEM. See Supplementary Table 2 for full statistical information. Source data are provided as a Source Data file.

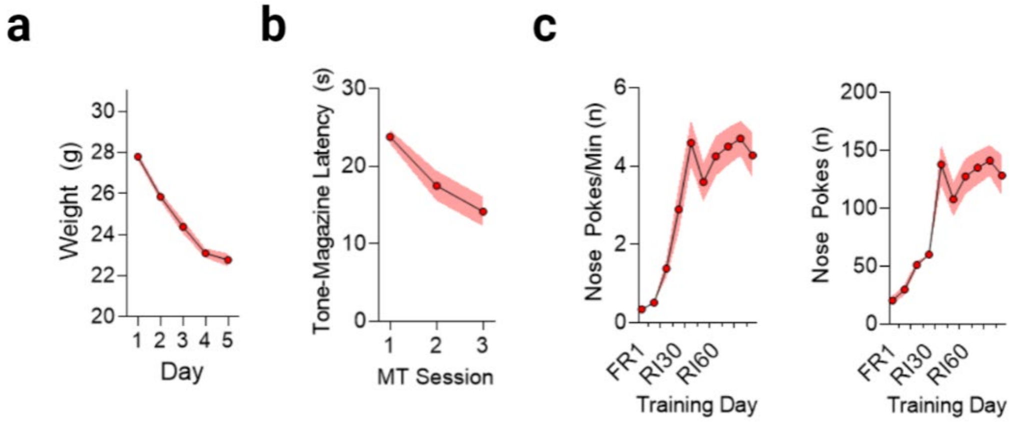

Supplementary Figure 9. Operant training of hM3Dq-expressing mice in external globus pallidus (GPe) arkypallidal neurons. (a) Average weight of mice undergoing food restriction prior to operant conditioning. **(b)** Latency to magazine decreased across training sessions. $F_{(1,49)} = 17.29$, $p = 0.0001$ for time. **(c)** Nose poke rates increased over time in the active hole. $F_{(1,168)} = 91.68$, $p < 0.0001$ for time. Total nosepokes for RI group across the training session. $F_{(1,168)} = 97.61$, $p < 0.0001$ for time. One-way ANOVA. $n = 17$ mice. Data represent mean ± SEM. See Supplementary Table 2 for full statistical information. Source data are provided as a Source Data file.

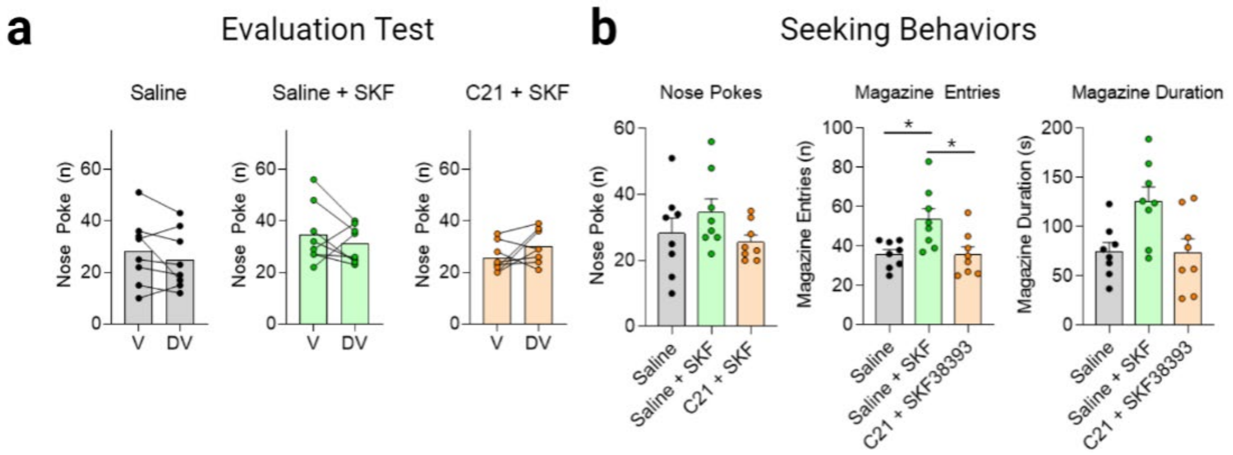

Supplementary Figure 10. Effect of D1R agonist, SKF38393, on RI operant conditioning. (a) No differences in nose poke rates between the valued and devalued state for RI-trained mice with saline, saline + SKF, nor C21 + SKF, indicating habitual reward-seeking. **(b)** Systemic D1R activation increased magazine entries compared to saline and coadministration of C21 in mice expressing hM3Dq in GPe arkypallidal neurons. For magazine entry, Dunn's *posthoc* tests show difference between Saline and Saline + SKF (p = 0.048), Saline + SKF and C21 + SKF (p = 0.034). Wilcoxon test was used for (**a**). Kruskal-wallis test with Dunn's *posthoc* tests were used for (**b**). n = 8 mice/group. Data represent mean ± SEM. *$p < 0.05$. See Supplementary Table 2 for full statistical information. Source data are provided as a Source Data file.

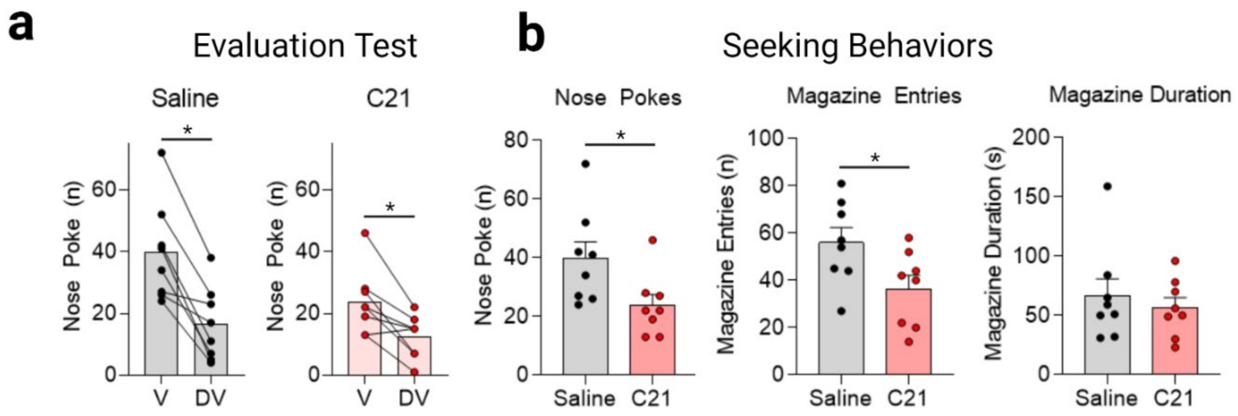

Supplementary Figure 11. Effect of chemogenetic activation of external globus pallidus (GPe) arkypallidal neurons on goal-directed RR conditioning. (a) Differences between the valued and devalued state for RR-trained mice with saline (p = 0.008) and C21 groups (p = 0.016), indicating goal-directed reward-seeking. **(b)** Chemogenetic activation of GPe arkypallidal neurons decreased nose poke (p = 0.03) and magazine entry (p = 0.031), but not in magazine duration (p = 0.67)-seeking behaviors during extinction testing. Wilcoxon test was used for (**a**). Two-tailed Mann Mann-Whitney tests were used for (**b**). n = 8 mice/group. Data represent mean ± SEM. *$p < 0.05$. See Supplementary Table 2 for full statistical information. Source data are provided as a Source Data file.

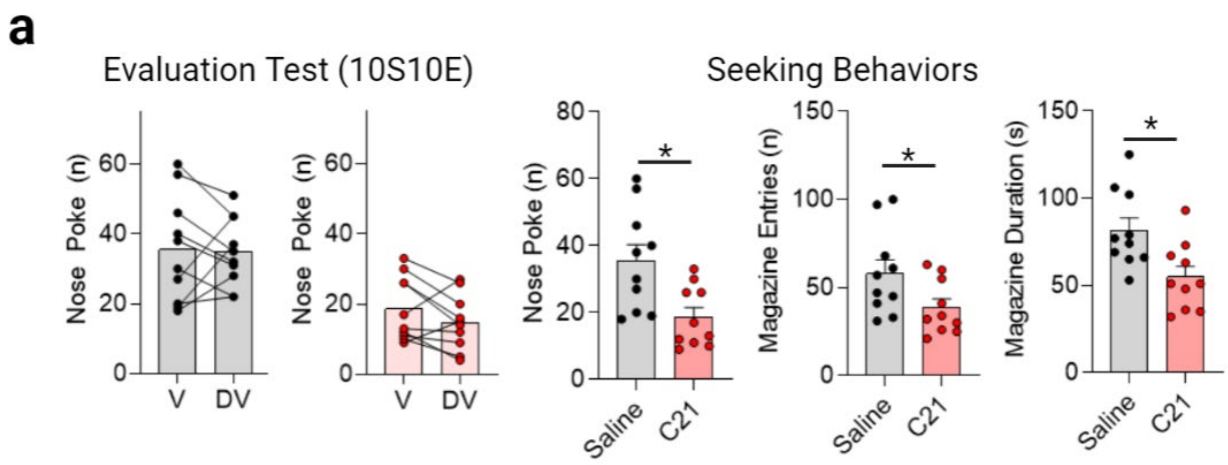

Supplementary Figure 12. Effect of chemogenetic activation of external globus pallidus (GPe) arkypallidal neurons on habitual sweetened ethanol-seeking. (a) No differences in nosepoke rates between the valued and devalued state for RI-trained mice with saline or C21 groups indicated habitual-seeking. **(b)** Chemogenetic activation of GPe arkypallidal neurons decreased nose poke (p = 0.0073), magazine entry (p = 0.046), and magazine duration (p = 0.0065)-seeking behaviors during extinction testing. Wilcoxon test was used for (**a**). Two-tailed Mann Mann-Whitney tests were used for (**b**). n = 10 mice/group. Data represent mean ± SEM. *p < 0.05. See Supplementary Table 2 for full statistical information. Source data are provided as a Source Data file.

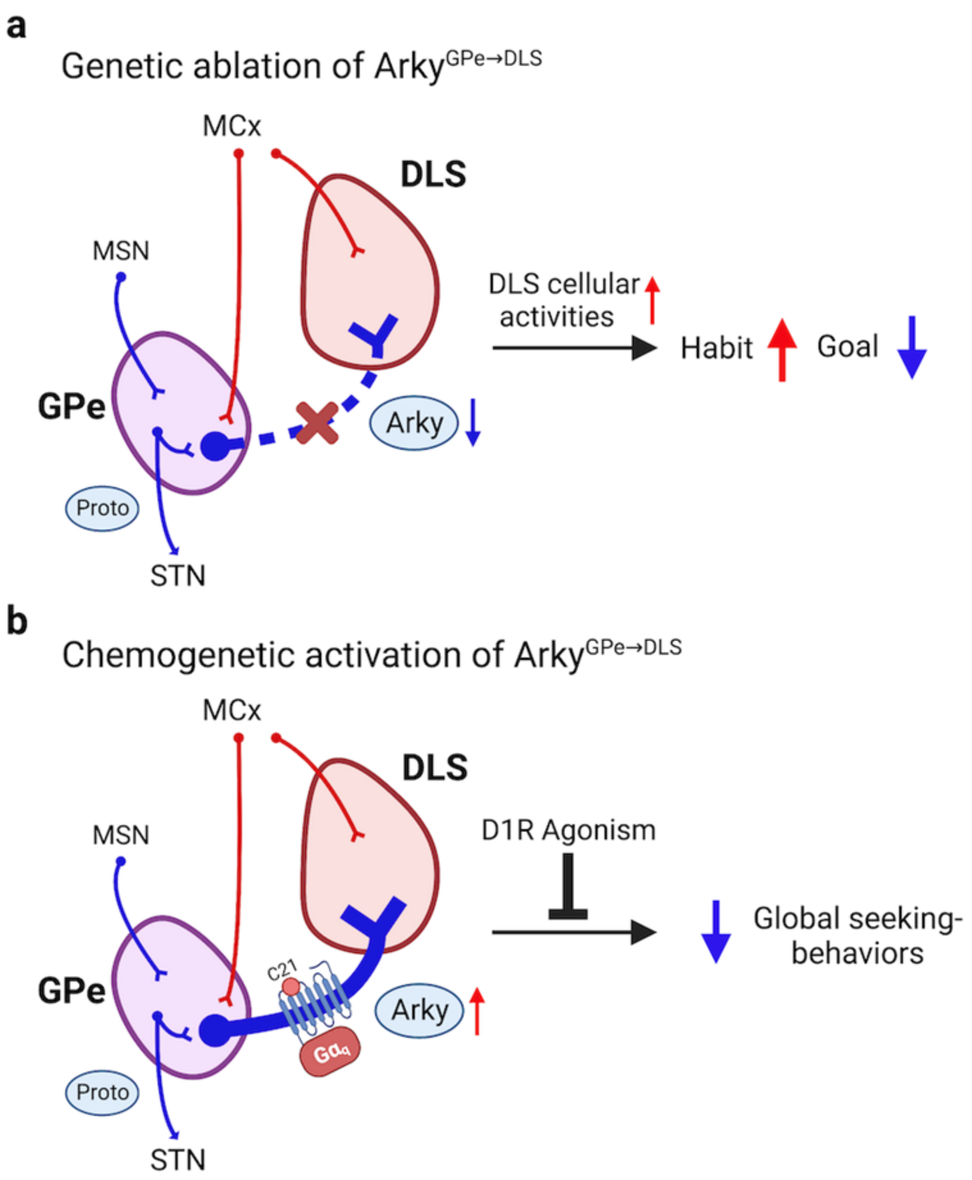

Supplementary Figure 13. Possible circuit mechanisms of GPe arkypallidal neurons on habitual behaviors. Circuit overview involving the external globus pallidus (GPe) and dorsolateral striatum (DLS), including excitatory glutamatergic (red) and inhibitory GABAergic (blue) projections. **(a)** Caspase 3-induced ablation of the arky$^{GPe\rightarrow DLS}$ circuit caused an increase in cFos expression in the DLS and resulted in a shift from goal-directed to habitual seeking. **(b)** In contrast, chemogenetic activation (hM3Dq) of arky$^{GPe\rightarrow DLS}$ caused a global reduction in seeking behaviors, which was blocked by D1R agonism. Possible afferent innervation of arkypallidal (arky) neurons from dorsal striatal medium spiny neurons (MSN), motor cortex (MCx), and neighboring prototypic (proto) neurons, which also project to the subthalamic nucleus (STN). Supplementary Figure 13a, and b were created with BioRender.com.

Supplementary Table 1. SVM AUC results to classify behavioral task types for all time windows of analysis using fiber photometry data.

Event	SVM Data input				AUC
	Before		After		
	Start (s)	End (s)	Start (s)	End (s)	
Nose-poke	-2	0	0	2	0.70 ± 0.001
	-1.5	0	0	1.5	0.68 ± 0.001
	-1	0	0	1	0.65 ± 0.001
	-0.5	0	0	0.5	0.65 ± 0.001
Magazine entry	-2	0	0	2	0.66 ± 0.001
	-1.5	0	0	1.5	0.67 ± 0.001
	-1	0	0	1	0.67 ± 0.001
	-0.5	0	0	0.5	0.68 ± 0.001
Magazine exit	-2	0	0	2	0.65 ± 0.001
	-1.5	0	0	1.5	0.68 ± 0.001
	-1	0	0	1	0.70 ± 0.001
	-0.5	0	0	0.5	0.71 ± 0.001

Supplementary Table 2. Summary of statistical analyses and results.

Figure	Statistical Tests	Comparison	Value	*p* value
Figure 1b	One-way ANOVA	Brain region	$F_{2,6}$ = 87.60	*p*<0.0001
	Tukey's posthoc	DMS vs DLS	q = 18.72	*p* = 0.0012
Figure 1d	Unpaired T-test	DMS vs DLS	t(4) = 8.72	*p* = 0.001
Figure 2b	Wilcoxon test	RR: V vs DV	W= -21.00	*p*= 0.030
		RI: V vs DV	W= -5.00	*p*= 0.62
Figure 2g	Two-way RM ANOVA	NP+: RR vs RI	$F_{1,7}$ = 5.73	*p*= 0.048
		NP+: Time	$F_{120,840}$ = 11.40	*p*< 0.0001
		NP+: Interaction	$F_{120,840}$ = 1.62	*p*= 0.0001
	Two-way RM ANOVA	NP-: RR vs RI	$F_{1,7}$ = 12.34	*p*= 0.0098
		NP-: Time	$F_{120,840}$ = 12.52	*p*< 0.0001
		NP-: Interaction	$F_{120,840}$ = 5.52	*p*< 0.0001
Figure 2h	Two-way RM ANOVA	ME+: RR vs RI	$F_{1,7}$ = 25.84	*p*= 0.0014
		ME+: Time	$F_{120,840}$ = 14.48	*p*< 0.0001
		ME+: Interaction	$F_{120,840}$ = 5.73	*p*< 0.0001
	Two-way RM ANOVA	ME -: RR vs RI	$F_{1,7}$ = 0.05	*p*= 0.83
		ME -: Time	$F_{120,840}$ = 5.60	*p*< 0.0001
		ME -: Interaction	$F_{120,840}$ = 1.74	*p*< 0.0001
Figure 3b	One-sample t-test	Accuracy NP vs 50%	t(1599) = 116.1	*p*< 0.0001
	One-sample t-test	Accuracy M_{Entry} vs 50%	t(1599) = 136.4	*p*< 0.0001
	One-sample t-test	Accuracy M_{Exit} vs 50%	t(1599) = 105.7	*p*< 0.0001
	One-sample t-test	Sensitivity NP vs 50%	t(1599) = 109.1	*p*< 0.0001
	One-sample t-test	Sensitivity M_{Entry} vs 50%	t(1599) = 126.9	*p*< 0.0001
	One-sample t-test	Sensitivity M_{Exit} vs 50%	t(1599) = 101.4	*p*< 0.0001
	One-sample t-test	Specificity NP vs 50%	t(1599) = 104.8	*p*< 0.0001
	One-sample t-test	Specificity M_{Entry} vs 50%	t(1599) = 118.9	*p*< 0.0001
	One-sample t-test	Specificity M_{Exit} vs 50%	t(1599) = 94.35	*p*< 0.0001
Figure 4c	Unpaired t-test	Control vs Casp3	t(8) = 6.90	*p* = 0.001
Figure 4e	Wilcoxon test	RR Control: V vs DV	W= -45.00	*p*= 0.0039
		RR Casp3: V vs DV	W= -7.00	*p*= 0.73
	Wilcoxon test	RI Control: V vs DV	W= -16.00	*p*= 0.38
		RI Casp3: V vs DV	W= 5.00	*p*= 0.83
Figure 4f	Unpaired T-test	DMS: Control vs Casp3	t(8) = 1.02	*p*= 0.34
	Unpaired T-test	DLS: Control vs Casp3	t(8) = 3.99	*p*= 0.004
Figure 5e	Paired T-test	Frequency: Veh vs C21	t(3) = 4.47	*p= 0.021*
	Paired T-test	Time: Veh vs C21	t(3) = 11.86	*p= 0.0013*
Figure 5f	Wilcoxon test	Saline: V vs DV	W= -58.00	*p*= 0.14
		C21: V vs DV	W= -21.00	*p*= 0.57

		C21+ Raclopride: V vs DV	W= -2.00	*p*= 0.91
		C21+ SKF38393: V vs DV	W= 0.00	*p*= 0.99
Figure 5g	Kruskal-Wallis test	Nose Poke: Drug		*p*= 0.0004
	Posthoc Dunn's test	Saline vs C21	Z = 2.71	*p*= 0.04
		Saline vs C21 + Raclopride	Z = 3.96	*p*= 0.0005
		Saline vs C21 + SKF38393	Z = 0.83	*p*= 0.99
	Kruskal-Wallis test	Magazine Entry: Drug		*p*< 0.0001
	Posthoc Dunn's test	Saline vs C21	Z = 3.59	*p*= 0.002
		Saline vs C21 + Raclopride	Z = 4.13	*p*= 0.0002
		Saline vs C21 + SKF38393	Z = 0.69	*p*= 0.99
Figure S2b	Two-way RM ANOVA	MT: Time	$F_{1,16}$ = 142.9	*p*<0.0001
		MT: Group	$F_{1,16}$ = 5.34	*p*= 0.040
		MT: Interaction	$F_{1,16}$ = 1.14	*p*= 0.30
Figure S2c	Two-way RM ANOVA	RR: NP	$F_{1,80}$ = 127.3	*p*< 0.0001
		RR: Time	$F_{9,80}$ = 4.99	*p*< 0.0001
		RR: Interaction	$F_{9,80}$ = 4.93	*p*< 0.0001
	Two-way RM ANOVA	RI: NP	$F_{1,80}$ = 142.1	*p*< 0.0001
		RI: Time	$F_{9,80}$ = 1.85	*p*= 0.070
		RI: Interaction	$F_{9,80}$ = 1.77	*p*= 0.090
Figure S2e	Two-way RM ANOVA	MX +: RR vs RI	$F_{1,7}$ = 6.66	*p*= 0.036
		MX +: Time	$F_{120,840}$ = 0.85	*p*= 0.87
		MX +: Interaction	$F_{120,840}$ = 1.26	*p*= 0.039
	Two-way RM ANOVA	MX -: RR vs RI	$F_{1,7}$ = 0.20	*p*= 0.67
		MX -: Time	$F_{120,840}$ = 8.65	*p*< 0.0001
		MX -: Interaction	$F_{120,840}$ = 5.51	*p*< 0.0001
Figure S3a	Linear Regression	NP+: RR	b= 0.065; R^2= .15; $F_{1,48}$= 8.26	*p*= 0.006
		NP+: RI	b= 0.10; R^2= .15; $F_{1,38}$= 6.78	*p*= 0.013
		NP+: RR vs RI	$F_{1,86}$= 0.79	*p*= 0.38
Figure S3b	Linear Regression	NP-: RR	b= 0.011; R^2= .03; $F_{1,48}$= 1.48	*p*= 0.23
		NP-: RI	b= 0.037; R^2= .23; $F_{1,38}$= 11.66	*p*= 0.002
		NP-: RR vs RI	$F_{1,86}$= 10.08	*p*= 0.002
Figure S3c	Linear Regression	ME+: RR	b= 0.076; R^2= .12; $F_{1,48}$= 6.62	*p*= 0.013
		ME+: RI	b= 0.18; R^2= .43; $F_{1,38}$= 28.86	*p*< 0.0001
		ME+: RR vs RI	$F_{1,86}$= 5.34	*p*= 0.023
Figure S3d	Linear Regression	ME-: RR	b= 0.044; R^2= .04; $F_{1,48}$= 1.96	*p*= 0.17
		ME-: RI	b= 0.080; R^2= .19; $F_{1,38}$= 9.08	*p*= 0.005
		ME-: RR vs RI	$F_{1,86}$= 0.69	*p*= 0.41
Figure S3e		MX+: RR	b= -0.08; R^2= .16; $F_{1,48}$= 9.08	*p*= 0.004

	Linear Regression	MX+: RI	b= -0.05; R^2= .08; $F_{1,38}$= 3.12	*p*= 0.09
		MX+: RR vs RI	$F_{1,86}$= 0.60	*p*= 0.44
Figure S3f	Linear Regression	MX-: RR	b= -0.06; R^2= .06; $F_{1,48}$= 3.30	*p*= 0.075
		MX-: RI	b= -0.12; R^2= .36; $F_{1,38}$= 21.77	*p*< 0.0001
		MX-: RR vs RI	$F_{1,86}$= 2.29	*p*= 0.13
Figure S4a	Mann-Whitney test	Distance: Control vs Casp3	U = 81	*p*= 0.45
		Velocity: Control vs Casp3	U = 91	*p*= 0.76
		Ambulatory Time: Control vs Casp3	U = 58	*p*= 0.069
		Ambulatory Episodes: Control vs Casp3	U = 97	*p*= 0.98
Figure S4b	Mann-Whitney test	Time in center: Control vs Casp3	U = 70	*p*= 0.21
		Time in periphery: Control vs Casp3	U = 70	*p*= 0.21
		Center Entries: Control vs Casp3	U = 80	*p*= 0.42
		Peripheral Entries: Control vs Casp3	U = 80	*p*= 0.42
Figure S4c	Two-Way RM ANOVA	Time	$F_{49,1274}$= 8.68	*p*< 0.0001
		Control vs Casp	$F_{1,26}$= 1.28	*p*= 0.27
		Interaction	$F_{49,1274}$= 0.76	*p*= 0.89
Figure S4d	Two-Way RM ANOVA	Time	$F_{4,130}$= 58.82	*p*< 0.0001
		Control vs Casp	$F_{1,130}$= 1.49	*p*= 0.22
		Interaction	$F_{4,130}$= 2.99	*p*= 0.018
	Posthoc Sidak test	Day 1	t(130) = 3.90	*p*= 0.0005
		Day 2	t(130) = 2.92	*p*= 0.018
		Day 3	t(130) = 1.11	*p*= 0.79
		Day 4	t(130) = 0.56	*p*= 0.98
		Day 5	t(130) = 1.25	*p*= 0.69
Figure S5b	Two-way RM ANOVA	RR MT: Time	$F_{1,18}$ = 29.91	*p*< 0.0001
		RR MT: Group	$F_{1,18}$ = 0.00	*p*= 0.99
		RR MT: Interaction	$F_{1,18}$ = 2.36	*p*= 0.11
	Two-way RM ANOVA	RI MT: Time	$F_{1,18}$ = 30.55	*p*< 0.0001
		RI MT: Group	$F_{1,18}$ = 0.13	*p*= 0.73
		RI MT: Interaction	$F_{1,18}$ = 0.01	*p*= 0.98
Figure S5c	Two-way RM ANOVA	RR: Time	$F_{9,161}$ = 13.38	*p*< 0.0001
		RR: Group	$F_{1,18}$ = 0.21	*p*= 0.65
		RR: Interaction	$F_{9,161}$ = 0.66	*p*= 0.74
	Two-way RM ANOVA	RI: Time	$F_{9,161}$ = 20.64	*p*< 0.0001
		RI: Group	$F_{1,18}$ = 0.28	*p*= 0.61
		RI: Interaction	$F_{9,161}$ = 0.52	*p*= 0.86
	Two-way RM ANOVA	RR: Time	$F_{9,161}$ = 18.29	*p*< 0.0001
		RR: Group	$F_{1,18}$ = 0.03	*p*= 0.86
		RR: Interaction	$F_{9,161}$ = 0.2	*p*= 0.99
	Two-way RM ANOVA	RI: Time	$F_{9,161}$ = 25.26	*p*< 0.0001
		RI: Group	$F_{1,18}$ = 0.17	*p*= 0.68

		RI: Interaction	$F_{9,161} = 0.35$	*p*= 0.96
Figure S6a	Wilcoxon test	RR Control: V vs DV	W= -43.00	*p*= 0.0078
		RR Casp3: V vs DV	W= 8.00	*p*= 0.72
Figure S6b	Wilcoxon test	RI Control: V vs DV	W= -28.00	*p*= 0.11
		RI Casp3: V vs DV	W= -29.00	*p*= 0.15
Figure S6c	Two-Way RM ANOVA (preference)	Time	$F_{13,234}= 9.09$	*p*< 0.0001
		Control vs Casp3	$F_{1,18}= 0.33$	*p*= 0.57
		Interaction	$F_{13,234}= 1.29$	*p*= 0.22
	Two-Way RM ANOVA (consumption)	Time	$F_{13,234}= 3.54$	*p*< 0.0001
		Control vs Casp3	$F_{1,18}= 0.39$	*p*= 0.54
		Interaction	$F_{13,234}= 0.87$	*p*= 0.58
Figure S7a	Kruskal-Wallace test	Distance traveled: C21 concentration		*p*= 0.28
		Velocity: C21 concentration		*p*= 0.29
		Ambulatory time: C21 concentration		*p= 0.56*
		Ambulatory episode: C21 concentration		*p= 0.37*
Figure S7b	Kruskal-Wallace test	Time in center: C21 concentration		*p= 0.17*
		Time in periphery: C21 concentration		*p= 0.17*
		Center entries: C21 concentration		*p= 0.37*
		Peripheral entries: C21 concentration		*p= 0.34*
Figure S8a	Kruskal-Wallace test	Distance traveled: IP injection groups		*p*= 0.07
		Velocity: IP injection groups		*p*= 0.42
		Ambulatory time: IP injection groups		*p= 0.19*
		Ambulatory episode: IP injection groups		*p= 0.02*
	Posthoc Dunn's test	Saline vs C21	Z = 0.05	p> 0.99
		Saline vs C21 + Raclopride	Z = 2.03	*p*= 0.13
		Saline vs C21 + SKF38393	Z = 0.79	*p*= 0.79
Figure S8b	Kruskal-Wallace test	Time in center: IP injection groups		*p= 0.30*
		Time in periphery: IP injection groups		*p= 0.30*
		Center entries: IP injection groups		*p= 0.68*
		Peripheral entries: IP injection groups		*p= 0.71*
Figure S9b	One-way RM ANOVA	MT Time	$F_{1,49}= 17.29$	*p*= 0.0001
Figure S9c	One-way RM ANOVA	NP rate: RI Time	$F_{1,168}= 91.68$	*p*< 0.0001

	One-way RM ANOVA	Total NP: RI Time	$F_{1,168}$= 97.61	*p*< 0.0001
Figure S10a	Wilcoxon test	Saline: V vs DV	W= -14.00	*p*= 0.36
		SKF: V vs DV	W= -14.00	*p*= 0.37
		SKF+C21: V vs DV	W = 19.00	*p*= 0.20
Figure S10b	Kruskal-Wallis test	Nose Poke: Drug		*p*= 0.26
	Kruskal-Wallis test	Magazine Entry: Drug		*p*= 0.02
	Posthoc Dunn's test	Saline vs SKF	Z = 2.41	*p= 0.048*
		Saline vs C21 + SKF	Z = 0.12	*p> 0.99*
		SKF vs C21 + SKF	Z = 2.53	*p*= 0.034
	Kruskal-Wallis test	Magazine Duration: Drug		*p*= 0.043
	Posthoc Dunn's test	Saline vs SKF	Z = 2.19	*p= 0.085*
		Saline vs C21 + SKF	Z = 0.04	*p> 0.99*
		SKF vs C21 + SKF	Z = 2.16	*p*= 0.093
Figure S11a	Wilcoxon test	Saline: V vs DV	W= -36.00	*p*= 0.008
		C21: V vs DV	W= -34.00	*p*= 0.016
Figure S11b	Mann-Whitney test	NP: Saline vs C21	U = 11.50	*p*= 0.03
		Entries: Saline vs C21	U = 11.50	*p*= 0.031
		Duration: Saline vs C21	U = 27.50	*p*= 0.67
Figure S12a	Wilcoxon test	Saline: V vs DV	W= -3.00	*p*= 0.90
		C21: V vs DV	W= -31.00	*p*= 0.13
Figure S12b	Mann-Whitney test	NP: Saline vs C21	U = 15.5	*p*= 0.0073
		Entries: Saline vs C21	U = 23.5	*p*= 0.046
		Duration: Saline vs C21	U = 15	*p*= 0.0065

Supplementary Table 3. Time range for significant (*posthoc* Sidak test; $p < 0.05$) individual comparisons between fiber photometry groups.

Figure	Comparison	Time range
Fig. 2g	NP+; RR vs. RI	NS
	NP-; RR vs. RI	0.73 – 1.7 s
Fig. 2h	M_{entry} +; RR vs. RI	0.00 – 2 s
	M_{entry} -; RR vs. RI	NS
Fig. S2e	M_{exit} +; RR vs. RI	NS
	M_{exit} -; RR vs. RI	0.03 – 0.27 s
