# Supplementary material for: External globus pallidus input to the dorsal striatum regulates habitual seeking behavior in male mice
Source: Nat Commun. 2023 Jul 12;14:4085. doi: 10.1038/s41467-023-39545-8 (PMC10338526; doi:10.1038/s41467-023-39545-8)
Supplement: Supplementary file 3 — Reporting Summary [file 41467_2023_39545_MOESM3_ESM.pdf]

nature portfolio

Corresponding author(s): Doo-Sup Choi

Last updated by author(s): 05/08/2023

Reporting Summary

Nature Portfolio wishes to improve the reproducibility of the work that we publish. This form provides structure for consistency and transparency in reporting. For further information on Nature Portfolio policies, see our Editorial Policies and the Editorial Policy Checklist.

Statistics

For all statistical analyses, confirm that the following items are present in the figure legend, table legend, main text, or Methods section.

n/a	Confirmed	
☐	☒	The exact sample size (*n*) for each experimental group/condition, given as a discrete number and unit of measurement
☐	☒	A statement on whether measurements were taken from distinct samples or whether the same sample was measured repeatedly
☐	☒	The statistical test(s) used AND whether they are one- or two-sided<br>*Only common tests should be described solely by name; describe more complex techniques in the Methods section.*
☒	☐	A description of all covariates tested
☐	☒	A description of any assumptions or corrections, such as tests of normality and adjustment for multiple comparisons
☐	☒	A full description of the statistical parameters including central tendency (e.g. means) or other basic estimates (e.g. regression coefficient) AND variation (e.g. standard deviation) or associated estimates of uncertainty (e.g. confidence intervals)
☐	☒	For null hypothesis testing, the test statistic (e.g. *F*, *t*, *r*) with confidence intervals, effect sizes, degrees of freedom and *P* value noted<br>*Give P values as exact values whenever suitable.*
☒	☐	For Bayesian analysis, information on the choice of priors and Markov chain Monte Carlo settings
☒	☐	For hierarchical and complex designs, identification of the appropriate level for tests and full reporting of outcomes
☒	☐	Estimates of effect sizes (e.g. Cohen's *d*, Pearson's *r*), indicating how they were calculated

Our web collection on statistics for biologists contains articles on many of the points above.

Software and code

Policy information about availability of computer code

Data collection	Med-Associates Med-PC software was used to collect behavioral data (version 4, Med Associates Inc.). Calcium imaging data was collected using CineLyzer software (version 1.0.1, Plexon Inc.). Some imaging data collected using ImageJ (version 1.53t, National Institute of Health, USA).
Data analysis	All data were processed using Microsoft Excel 2013 (Redmond, WA), then compiled and statistically analyzed with GraphPad Prism v9.0 (La Jolla, CA). Custom MATLAB (version R2020a) script was used for SVM modeling/analysis (MATLAB code is provided at https://github.com/brain-machine-intelligence/Baker_2022 and referenced in the Code Availability Statement).

For manuscripts utilizing custom algorithms or software that are central to the research but not yet described in published literature, software must be made available to editors and reviewers. We strongly encourage code deposition in a community repository (e.g. GitHub). See the Nature Portfolio guidelines for submitting code & software for further information.

Data

Policy information about availability of data

All manuscripts must include a data availability statement. This statement should provide the following information, where applicable:
- Accession codes, unique identifiers, or web links for publicly available datasets
- A description of any restrictions on data availability
- For clinical datasets or third party data, please ensure that the statement adheres to our policy

The data generated in this study are provided in the Source Data file.

Human research participants

Policy information about studies involving human research participants and Sex and Gender in Research.

Reporting on sex and gender	not applicable.
Population characteristics	not applicable.
Recruitment	not applicable
Ethics oversight	not applicable

Note that full information on the approval of the study protocol must also be provided in the manuscript.

Field-specific reporting

Please select the one below that is the best fit for your research. If you are not sure, read the appropriate sections before making your selection.

☒ Life sciences ☐ Behavioural & social sciences ☐ Ecological, evolutionary & environmental sciences

For a reference copy of the document with all sections, see nature.com/documents/nr-reporting-summary-flat.pdf

Life sciences study design

All studies must disclose on these points even when the disclosure is negative.

Sample size	Animal numbers were determined by pilot data and previous studies in our laboratory, primarily Kang et. al (2020; 10.1016/j.biopsych.2020.04.023). These informed a power analysis using a power value of 0.8 and an alpha of 0.05.
Data exclusions	one animal was excluded from calcium imaging due to loss of head cap
Replication	Calcium imaging data was completed with RI first (n = 5) followed by RR (n = 5). The caspase data was performed in two cohorts, RI control/casp3 first (n =10/group), followed by RR control/casp3 (n = 10/group). Then the replicate using 10% ethanol/10% sucrose reward was completed in one cohort (n = 8/group). DREADD data was completed in two cohorts (n = 9, then n = 8, 17 total). Then the replicate using 10% ethanol/10% sucrose reward was completed in a second cohort (n = 8).
Randomization	all animals were randomly assigned into the experimental and control groups. Behavioral experiments and drug injections order were counterbalanced.
Blinding	investigators were not blinded to pharmacological or chemogenetic experiments because they were required to administer the drug. All behavioral measures were recorded automatically using Med-PC systems.<br><br>Behavioral experimenter was blinded to the viral injection groups.

Reporting for specific materials, systems and methods

We require information from authors about some types of materials, experimental systems and methods used in many studies. Here, indicate whether each material, system or method listed is relevant to your study. If you are not sure if a list item applies to your research, read the appropriate section before selecting a response.

Materials & experimental systems

n/a	Involved in the study
☐	☒ Antibodies
☒	☐ Eukaryotic cell lines
☒	☐ Palaeontology and archaeology
☐	☒ Animals and other organisms
☒	☐ Clinical data
☒	☐ Dual use research of concern

Methods

n/a	Involved in the study
☒	☐ ChIP-seq
☒	☐ Flow cytometry
☒	☐ MRI-based neuroimaging

Antibodies

Antibodies used	Primary: anti-FOXP2 (ab16046), 1:500, abcam, Cambridge, UK; anti-parvalbumin antibody (ab11427), 1:500, abcam. Secondary: anti-rabbit Alexa Fluor405 (donkey; ab17561; 1:500)
Validation	abcam antibodies have been validated by the manufacturer using immunofluorescence and immunoprecipitation. Relevant citations can be found on the manufacturers website for anti-FOXP2 (https://www.abcam.com/foxp2-antibody-ab16046.html) and anti-parvalbumin (https://www.abcam.com/parvalbumin-antibody-ab11427.html)

Animals and other research organisms

Policy information about studies involving animals; ARRIVE guidelines recommended for reporting animal research, and Sex and Gender in Research

Laboratory animals	Male, C57BL/6 mice (aged 8-10 weeks at beginning of experiments; Jackson Laboratory, Bar Harbor, ME) were used.
Wild animals	The study did not involve wild animals.
Reporting on sex	Experiments were conducted using male C57BL/6 mice. Limitations and future directions were discussed in the text regarding sex-specific analyses.
Field-collected samples	The study did not involve samples collected from the field.
Ethics oversight	All experimental procedures were approved by the Mayo Clinic Institutional Animal Care and Use Committee and performed following NIH guidelines.

Note that full information on the approval of the study protocol must also be provided in the manuscript.
